# Investigating the factor structure and measurement invariance of the eating disorder examination questionnaire (EDE-Q) among cisgender gay men and lesbian women from the United States

**DOI:** 10.1186/s40337-023-00880-2

**Published:** 2023-09-22

**Authors:** Emilio J. Compte, F. Hunter McGuire, Tiffany A. Brown, Jason M. Lavender, Stuart B. Murray, Matthew R. Capriotti, Annesa Flentje, Micah E. Lubensky, Mitchell R. Lunn, Juno Obedin-Maliver, Jason M. Nagata

**Affiliations:** 1 Eating Behavior Research Center, School of Psychology, Universidad Adolfo Ibáñez, 2640 Diagonal Las Torres Avenue, Peñalolén, Santiago Chile; 2 Research Department, Comenzar de Nuevo Treatment Center, Av. Humberto Lobo 1001, Del Valle, 66220 San Pedro Garza García, N.L. Mexico; 3 The Brown School, Washington University in St. Louis, 1 Brookings Dr, St. Louis, MO 63130 USA; 4 Department of Psychological Sciences, Auburn University, 101 Cary Hall, Auburn, AL 36849-5234 USA; 5 Military Cardiovascular Outcomes Research Program (MiCOR), Department of Medicine, Uniformed Services University of the Health Sciences, 4301 Jones Bridge Rd, Bethesda, MD 20814 USA; 6 The Metis Foundation, 84 NE Interstate 410 Loop # 325, San Antonio, TX 78216 USA; 7 Department of Psychiatry and Behavioral Sciences, University of Southern California, 1975 Zonal Ave., Los Angeles, CA 90033 USA; 8 Department of Psychology, San José State University, 1 Washington Sq, San Jose, CA 95192 USA; 9 The PRIDE Study/PRIDEnet, Stanford University School of Medicine, 291 Campus Drive Li Ka Shing Building, Stanford, CA 94305-5101 USA; 10 Department of Community Health Systems, University of California, San Francisco, 675 18th St. UCSF Pritzker Psychiatry Building, San Francisco, CA 94107 USA; 11 Alliance Health Project, Department of Psychiatry and Behavioral Sciences, University of California, San Francisco, 401 Parnassus Ave, San Francisco, CA 94143 USA; 12 Division of Nephrology, Department of Medicine, Stanford University School of Medicine, 300 Pasteur Drive, Stanford, CA 94304 USA; 13 Department of Epidemiology and Population Health, Stanford University School of Medicine, 300 Pasteur Drive, Stanford, CA 94305 USA; 14 Department of Obstetrics and Gynecology, Stanford University School of Medicine, 770 Welch Road, #201, Palo Alto, CA 94304 USA; 15 Department of Pediatrics, University of California, San Francisco, 550 16th Street, 4th Floor, Box 0503, San Francisco, CA 94143 USA

**Keywords:** Eating disorders, Assessment, Transgender, Transmasculine, Transfeminine, Non-binary, Gender minority, Sexual and gender minority, LGBTQ+, Eating disorder examination-questionnaire

## Abstract

**Background:**

Although the Eating Disorder Examination-Questionnaire (EDE-Q) is one of the most widely used self-report assessments of eating disorder symptoms, evidence indicates potential limitations with its original factor structure and associated psychometric properties in a variety of populations, including sexual minority populations. The aims of the current investigation were to explore several previously published EDE-Q factor structures and to examine internal consistency and measurement invariance of the best-fitting EDE-Q model in a large community sample of cisgender gay men and cisgender lesbian women.

**Methods:**

Data were drawn from 1624 adults (1060 cisgender gay men, 564 cisgender lesbian women) who participated in The PRIDE Study, a large-scale longitudinal cohort study of sexual and gender minorities from the United States. A series of confirmatory factor analyses (CFAs) were conducted to explore the fit of eight proposed EDE-Q models; internal consistency (Cronbach’s alphas, Omega coefficients) and measurement invariance (multi-group CFA) were subsequently evaluated.

**Results:**

A brief seven-item, three-factor (dietary restraint, shape/weight overvaluation, body dissatisfaction) model of the EDE-Q from Grilo et al. (Obes Surg. 23:657–662, 2013), consistently evidenced the best fit across cisgender gay men and lesbian women. The internal consistencies of the three subscales were adequate in both groups, and measurement invariance across the groups was supported.

**Conclusions:**

Taken together, these findings support the use of the seven-item, three-factor version of the EDE-Q for assessing eating disorder symptomatology in cisgender gay men and lesbian women. Future studies can confirm the current findings in focused examinations of the seven-item, three-factor EDE-Q in diverse sexual minority samples across race, ethnicity, socioeconomic status, and age ranges.

## Abstract (plain language summary)

We asked cisgender gay men and lesbian women in The PRIDE Study to fill out a widely used survey about eating disorders, the Eating Disorder Examination-Questionnaire. We found that a version of this questionnaire based on seven questions including three parts—(1) dietary restraint, (2) shape and weight overvaluation, and (3) body dissatisfaction—had the best fit. These findings can assist doctors and scientists in understanding eating disorders in cisgender gay men and lesbian women.

## Introduction

Extant literature suggests that cisgender (i.e., those whose gender is what is commonly associated with the sex assigned to them at birth) sexual minority (e.g., those with a gay, lesbian, bisexual, pansexual, queer, or other non-heterosexual identity) individuals are more likely than their cisgender heterosexual counterparts to experience eating disorders and engage in disordered eating behaviors [1]. Mechanisms proposed to explain sexual orientation disparities in mental health have most often centered on the adverse impacts of systems of oppression (e.g., heterosexism) and minority stress processes. Minority stress theory posits that social, economic, and political forces engender environmental contexts that disproportionately expose cisgender gay men and lesbian women to distal (e.g., discrimination, violence, harassment) and proximal (e.g., internalized homophobia) stressors [2, 3]. These stressors, in turn, contribute to a greater risk for adverse health outcomes, including eating disorders and disordered eating, in sexual minority populations.

Among sexual minority people, there is notable heterogeneity in the prevalence of eating disorder symptomatology and related risk and protective factors [1, 4]. For instance, prior literature consistently finds a higher prevalence of eating disorders and related behaviors among sexual minority men versus heterosexual men, whereas findings from comparisons of sexual minority women and heterosexual women have been more mixed in terms of the direction and magnitude of potential differences [1]. This disparate pattern of results across specific sexual minority groups may be driven by a diverse array of subgroup-specific stressors and protective factors, including cultural norms de-emphasizing thin-ideal body image standards among lesbian women [5, 6] and heightened focus on physical appearance and attractiveness in gay male communities [7]. Given the evidence of group differences among cisgender sexual minority people [8, 9], ensuring that eating disorder assessment measures are appropriate and valid for use with specific sexual minority groups is critical for properly evaluating the nature and severity of eating disorder symptoms for these potentially at-risk populations.

The Eating Disorder Examination-Questionnaire (EDE-Q) [10] is a self-report measure that is commonly used in research and clinical settings; however, the original four-factor structure (Restraint, Eating Concern, Weight Concern, Shape Concern) of the EDE-Q generally does not replicate across samples, and a variety of alternative factor structures have been empirically supported in various populations [11–17]. For example, an alternative four-factor structure was identified by Friborg et al. in a community sample of Norwegian women: one factor consisting of mostly Weight Concern and Shape Concern items, one factor with a mix of Restraint and Eating Concern items, one factor with only Eating Concern items, and one factor with only Restraint items [13]. In an exploratory factor analysis with a sample of US adult women, Peterson et al. identified a three-factor structure with most items from Weight Concern and Shape Concern subscales loading onto one factor [16]. Among Fijian adolescent girls, Becker et al. found support for a modified two-factor structure where most items from the Shape Concern, Weight Concern, and Eating Concern subscales loaded onto a single factor [11]. Prior research has identified single-factor solutions, including a 22-item model based on a sample of Dutch women [15] and a brief eight-item model with only Weight Concern and Shape Concern items among US adolescent girls [17] and eating disorder treatment-seeking US adult women [12]. Prior research has also compared the original full-length version of the EDE-Q and several proposed short forms (7-item, 8-item, and 18-item) in terms of their respective psychometric properties [18]. Notably, a brief seven-item, three-factor version (Dietary Restraint, Shape/Weight Overvaluation, and Body Dissatisfaction) that was originally identified by Grilo et al. in a sample of patients presenting for bariatric surgery [14] has subsequently been replicated in numerous studies of diverse populations including gender minority (e.g., transgender and gender-expansive) adults in the US [19]. Measurement invariance and external validity of this brief seven-item EDE-Q also has been supported in a sample of patients seeking bariatric surgery who identified as Latinx and were English- or Spanish-speaking [20].

There have been relatively few investigations of the psychometric properties of the EDE-Q in sexual minority populations. A study of cisgender gay and bisexual adult men in Brazil found support for a one-factor structure with 22 items [21]. To our knowledge, only one prior study has assessed the factor structure and psychometric properties of the EDE-Q among cisgender sexual minority people living in the US [22]. Klimek et al. tested the original four-factor structure of the EDE-Q [10] and two alternatives proposed by Friborg et al. and Grilo et al. [13, 14]. Results indicated that the alternative models outperformed the original model; there was adequate internal consistency for all subscales of alternative models, and such alternative models supported gender-based measurement invariance. However, this study was limited in two critical ways. First, participants were aged 18–30 years, thus limiting generalizability with regard to sexual minority adults in middle and older age groups. Second, and most importantly, non-heterosexual individuals of diverse sexual identities (e.g., lesbian or gay, bisexual, queer) were combined to form two gender-based sexual minority groups (i.e., sexual minority women versus sexual minority men). Due to the diverse stressors and experiences across specific sexual minority communities as described above (e.g., biphobic discrimination [1]), it is unclear whether results from this study would be replicated when specifically examining cisgender gay men and lesbian women.

The current study reports on cisgender gay men and lesbian women recruited from The Population Research in Identity and Disparities for Equality (PRIDE) Study, a cohort study of sexual and gender minority adults in the US. By including a broader age range (18–80 years old) and examining specific sexual minority subgroups, as well as examining a wider array of potential factor structures, this study serves as both a replication and an extension of Klimek et al. [22]. Our aims were to (1) test several previously investigated EDE-Q factor structures and identify the best-fitting model separately in samples of cisgender gay men and lesbian women; and (2) for the best-fitting model, evaluate internal consistency and measurement invariance across the two groups.

## Methods

### Participants

The PRIDE Study is a national (US), longitudinal, cohort study of sexual and/or gender minority adults, including individuals who identify as lesbian, gay, bisexual, transgender, and queer (LGBTQ), or another sexual and/or gender minority identity. Specific inclusion criteria included: identifying as a sexual and/or gender minority person, living in the US or its territories, being at least 18 years old, and being able to read and respond to questionnaires written in English. Data were collected with a bespoke, digital research platform accessible from any computer, tablet, or smartphone. Participants were recruited through PRIDEnet (a national network of organizations and individuals to engage LGBTQ + communities in health research), newsletters and blog posts, distribution of promotional items, outreach at conferences and events, social media advertising, and word-of-mouth. Additional details about The PRIDE Study research recruitment, platform, and design have been described elsewhere [24, 25].

A total of 4285 participants in The PRIDE Study completed the ‘Eating and Body Image’ survey from which the current data were collected. Of those, 1,090 were classified as cisgender gay men, and 564 were classified as cisgender lesbian women. We excluded 30 cisgender gay men and 36 cisgender lesbian women from analyses due to the presence of > 50% of missing values as data with over 50% missingness may be questionable [26]; thus, the overall sample used for analyses included 1060 gay men and 528 lesbian women. For missing data < 50%, data imputation was performed using multivariate imputation by chained equations. This method was chosen because, for all cases, the underlying mechanism of missingness was consistent with missing completely at random according to the non-parametric test of homoscedasticity (*p*s > 0.050) [27].

### Cisgender gay men

Cisgender gay men (*n* = 1060) had a mean age of 42.1 years (*SD* = 15.1, range = 18–82) and a mean body mass index of 27.2 kg/m^2^ (*SD* = 6.2, range = 16.1–64.9). In terms of race, 76.4% identified as White, 1.7% as Black, 2.9% as Asian, 0.6% as Native American, 3.9% as another race, 4.3% as multi-race, and 10.2% did not provide data on race. In addition, 3.9% identified as Hispanic. Further, 73.3% of cisgender gay men had a college education or higher, 93.7% were born in the US, and 2.9% reported being told by a healthcare provider that they had an eating disorder.

### Cisgender lesbian women

Cisgender lesbian women (*n* = 564) had a mean age of 38.3 (*SD* = 14.4, range = 18–77) and a mean body mass index of 29.1 kg/m^2^ (*SD* = 8.2, range = 16.6–67.9). In terms of race, 74.5% identified as White, 1.4% as Black, 1.1% as Asian, 0.4% as Native American, 6.1% as another race, 3.7% as multi-race, and 12.8% did not provide data on race. In addition, 5.3% identified as Hispanic. Further, 73.7% of cisgender lesbian women had a college education or higher, 92.9% were born in the US, and 7.1% reported being told by a healthcare professional that they had an eating disorder.

### Data analysis

Assumptions of multivariate normality were not fulfilled for both cisgender gay men and cisgender lesbian women. Given this and the ordinal nature of the data, the confirmatory factor analyses (CFAs) were based on diagonally weighted least squares (WLSMV) estimation method, as it is less biased than other robust methods [28]. To adequately evaluate model fit, we considered several indices including: Comparative Fit Index (CFI), Root Mean Square Error of Approximation (RMSEA) and its 90% confidence interval, and the Weighted Root Mean Square Residual (WRMR). Following Hu and Bentler (1999) [29] and Distefano et al. (2018) [30], model fit was determined via consensus among these three indices: CFI values ≥ 0.95, SRMR values ≤ 0.08, and RMSEA values ≤ 0.06 suggest a good fit of the model to the data, whereas CFI values 0.90–0.94, SRMR values 0.09–0.10, and RMSEA values 0.07–0.10, and WRMR ≤ 1.0, suggest an acceptable fit.

We investigated the following EDE-Q factor structures: *Model 1*: the original 22-item 4-factor model (Restraint, Eating Concern, Weight Concern, and Shape Concern) described by Fairburn and Beglin [31]; *Model 2*: a three-factor model that retains two EDE-Q subscales (Restraint, Eating Concern) but combines Weight and Shape Concern items [16]; *Model 3*: a two-factor model that retains the EDE-Q Restraint subscale and collapses Eating, Weight, and Shape Concern items into a second subscale [11]; *Model 4*: a 22-item one-factor model [15]; *Model 5*: A brief eight-item one-factor model comprising Weight and Shape Concern items [12, 17]; *Model 6*: an alternative 22-item four-factor model described by Friborg et al. (2013) [13]: *Model 7*: a 22-item four-factor model based on Friborg et al.’s (2013) model but including a general latent ‘*g*’ factor accounting for the variance in all items; and *Model 8*: a brief seven-item three-factor model (Dietary Restraint, Shape/Weight Overvaluation, and Body Dissatisfaction) described by Grilo et al. [14]. The Expected Cross Validation Index (ECVI) was assessed for model comparison with lower values representing a combination of a better fit to the data and a more parsimonious model. The ECVI index is preferred over other non-nested model comparison indexes (e.g., the Akaike Information Criterion (AIC)) [32]) since it “also incorporates sample size—specifically, a greater penalty function for fitting a non-parsimonious model in a smaller sample” [33].

For the model that was found to evidence the best fit in the current samples, internal consistency was assessed through Cronbach’s alpha and the Omega coefficient [34, 35]. However, following recommendations by Eisinga et al. [36], internal consistency for 2-item factors was conducted using the Spearman-Brown reliability coefficient. Internal consistency values > 0.80 were considered adequate [35, 37]. Finally, a multi-group CFA/measurement invariance analysis was conducted [38] for the best-fitting model to assess configural, metric, and scalar invariance across the sexual minority groups (i.e., gay men and lesbian women). Briefly, configural invariance assumes that the hypothesized factor structure is equivalent across groups (if data does not fit at this level, invariance does not hold at any level). Metric invariance implies that factor loading magnitudes are equal, and scalar invariance denotes that item loadings and intercepts are similar. ΔCFI < 0.01 is broadly considered as an indicator of metric invariance (non-significant Δ ${\chi }^{2}$ were also expected for metric invariance, as it implies that the invariance model is a better representation of the data), and scalar invariance is supported when ΔCFI < 0.01 and ΔRMSEA < 0.015 [38, 39].

R software (version 3.4.4) and the following packages were used: *MissMech* [27]; *Mice* [40]; *MVN* [41]; *Lavaan* [42], semTools [43]; *MBESS* [44]; *Psych* [45] and *Hmisc* [46].

## Results

### Factor structure evaluation

**Fig. 1:**
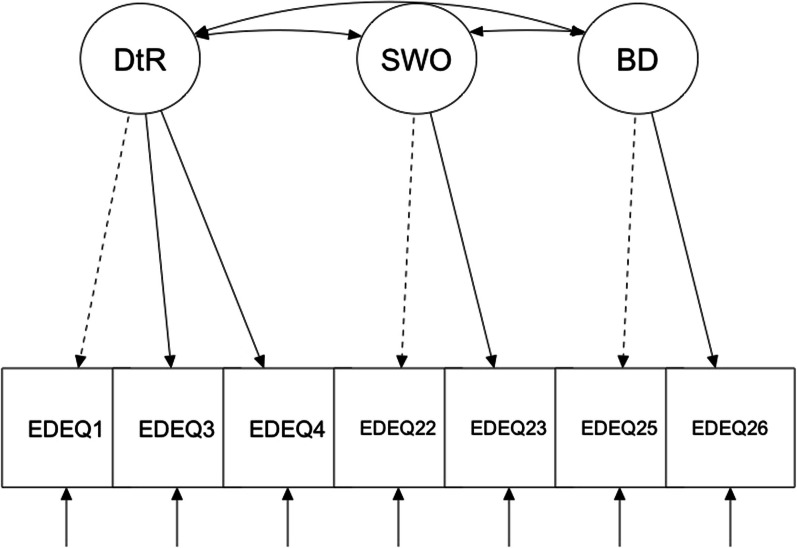
Conceptual representation of Grilo et al.’s Brief 3-factor 7-item EDE-Q. DtR = Dietary Restraint; SWO = Shape/Weight Overvaluation, BD = Body Dissatisfaction. Dashed lines indicate the factor loading for that item was fixed to 1 to ensure an identified model

A series of CFAs on the previously described models across sexual minority participants are shown in Table 1. *Model 1* assessed the 4-factor 22-item original proposal by [10, 31]; however, results revealed a nonpositive definite matrix solution, suggesting that this model was unacceptable. For *Models 2, 3, 6, and 7,* CFI and WRMR values were not adequate for either cisgender gay men or lesbian women, while RMSEA and SRMR values were adequate. Moreover, values for all three fit indices were inadequate for *Model 4*. *Model 5* demonstrated adequate CFI and SRMR values among cisgender gay men and cisgender lesbian women. WRMR values for gay men were above the suggested threshold but adequate for lesbian women, but RMSEA values exceeded the suggested threshold, in both cisgender gay men and cisgender lesbian women. Finally, in both groups, *Model 8* showed an excellent fit to the data and had the lowest ECVI values across models. As such, *Model 8* was retained for both cisgender gay men and cisgender lesbian women (Fig. 1).

**Table 1:** Confirmatory Factor Analyses of the tested models

EDE-Q models		Fit indices & model comparison index			
		CFI	RMSEA (CI 90%)	WRMR	ECVI
Model 2. 3-factor model					
	Gay men (n = 1060)	.83	.08 (.08, .09)	1.90	0.95
	Lesbian women (n = 528)	.79	.09 (.08, .09)	1.48	1.23
Model 3. 2-factor model					
	Gay men (n = 1060)	.83	.08 (.08, .09)	2.02	1.06
	Lesbian women (n = 528)	.79	.09 (.08, .09)	1.54	1.30
Model 4. Full 1-factor model					
	Gay men (n = 1060)	.71	.11 (.10, .11)	2.60	1.69
	Lesbian women (n = 528)	.70	.11 (.10, .11)	1.81	1.73
Model 5. Brief 8-item model					
	Gay men (n = 1060)	.94	.12 (.11., 13)	1.34	0.09
	Lesbian women (n = 528)	.94	.12 (.10, .14)	0.94	0.12
Model 6. 4-factor model					
	Gay men (n = 1060)	.86	.08 (.07, .08)	1.66	0.75
	Lesbian women (n = 528)	.86	.08 ( .07, .08)	1.66	0.75
Model 7. Bi factor model (4 factors and a general factor)					
	Gay men (n = 1060)	.85	.08 (.07, .08)	1.75	0.82
	Lesbian women (n = 528)	.80	.09 (.08, .09)	1.41	1.14
Model 8. Brief 3-factor 7-item model					
	Gay men (n = 1060)	.99	.06 (.05, .08)	0.76	0.05
	Lesbian women (n = 528)	.98	.07 (.05, .10)	0.62	0.09

*CFI* Comparative Fit Index; *RMSEA* root mean square error of approximation; *WRMR* weighted root mean square residual; *ECVI* expected cross validation index

### Internal consistency

Adequate internal consistency was found for the three subscales of the retained model in both sexual minority groups. For the three-item dietary restraint subscale, Cronbach’s alpha values were 0.85 for cisgender gay men and 0.84 for cisgender lesbian women. The omega coefficient values for the subscale were 0.85 (95% CI 0.83, 0.87) for cisgender gay men and 0.84 (95% CI 0.81, 0.87) for cisgender lesbian women. For the two-item Shape/Weight Overvaluation subscale, the Spearman-Brown coefficient was 0.91 for cisgender gay men and 0.95 for cisgender lesbian women. Finally, for the two-item Body Dissatisfaction subscale, the Spearman-Brown coefficient values were 0.89 for cisgender gay men and 0.91 for cisgender lesbian women.

### Measurement invariance

Table 2 shows results from a multi-group CFA that was conducted to evaluate measurement invariance of *Model 8* (i.e., the retained brief seven-item three-factor model that showed evidence of the best fit in both groups). Measurement invariance among cisgender gay men and cisgender lesbian women was supported at the configural level, indicating that the number of latent factors and the pattern of item loadings were similar across the two sexual minority groups. In addition, metric invariance was observed ($\Delta$ CFI = 0.004), indicating that the magnitude of the loadings was similar across the two groups. Consistently, the non-significant Δ ${\chi }^{2}$ supported metric invariance. Further, scalar invariance was observed (ΔCFI = -0.005 and ΔRMSEA = 0.006), indicating that item loadings and item intercepts were similar across groups (Table 2). Finally, a CFA of the retained brief 7-item 3-factor model (*Model 8*) including all participants (cisgender gay men and lesbian women; *N* = 1,588) resulted in adequate fit to the data (CFI = 0.98, RMSEA = 0.07 [90% CI = 0.05, 0.08], SRMR = 0.02).

**Table 2:** Measurement invariance between subset samples of cisgender gay men (n = 1060) and cisgender lesbian women (n = 528) from The PRIDE Study

Fit	X2	*df*	CFI	RMSEA	Model Comparison	∆X2	∆ CFI	∆ RMSEA	∆ *df*	*p*
Configural	96,628	22	.984	.065	–	–	–	–	–	
Metric	82,137	26	.988	.052	Configural vs. Metric	–14.491	.004	−.013	4	.597
Scalar	110,801	30	.983	.058	Metric vs. Scalar	28,664	−.005	.006	4	< .001

*CFI* comparative fit index; *RMSEA* root mean square error of approximation

## Discussion

In a large US-based sample reflecting a wide age range of cisgender gay men and lesbian women, we used a comparative CFA approach to evaluate various models of the EDE-Q that have been supported across diverse samples in prior research. To help inform future research in this area, we subsequently explored measurement invariance of the best-fitting model. Results suggested that a seven-item, three-factor model identified by Grilo et al. [14] had the best fit in both samples. Further, the subscales (Dietary Restraint, Shape/Weight Overvaluation, and Body Dissatisfaction) of this brief version of the EDE-Q were found to have adequate internal consistency reliability in cisgender gay men and lesbian women, and results supported measurement invariance of the three-factor model across the sexual minority groups.

The current results from our comparative CFA-based evaluation of EDE-Q factor structures are consistent with prior research on cisgender sexual minority adults [22] and extend these results to specific samples of cisgender gay men and cisgender lesbian women reflecting an age range from young to late adulthood. Notably, the original four-factor structure of the EDE-Q was not supported in the present study nor in Klimek et al. [22], further suggesting that using the EDE-Q in its standard form may not be appropriate for cisgender sexual minority individuals. Results supported measurement invariance comparing gay men and lesbian women in the present sample, suggesting that the three-factor EDE-Q model measures the same construct of eating disorder psychopathology across these groups. Further, scalar invariance results indicate that future research using this three-factor version can validly compare mean scores across gay men and lesbian women, which will support research examining similarities and differences in symptoms, risk, and disparities (compared to heterosexual peers) across these groups. Of note, the present study only included cisgender gay men and lesbian women and did not include individuals who identify as bisexual or any other sexual orientation. Given that bisexual individuals may differ from gay men and lesbian women with regard to experiences of harassment and discrimination (e.g., biphobic discrimination [8]), as well as prior research suggesting that eating disorder symptoms and severity may differ across sexual orientation subgroups [1], future research should aim to explore differences in samples of individuals with other specific sexual orientation identities such as bisexual people.

An additional consideration regarding the current findings is that the seven-item, three-factor EDE-Q model supported in this study includes no items from the original Eating Concern subscale and has two subscales with only two items, which could produce unreliable reporting in small samples. However, despite the small number of items, the internal consistencies (indexed by correlations) for these subscales were high in both sexual minority groups. Moreover, this briefer EDE-Q may be well-suited as a screening measure, which could be ideal in primary care settings, outpatient clinics, or other circumstances in which time is especially limited. Further, shortened versions can help reduce overall assessment burden, particularly in the context of a broader clinical evaluation or research study that involves completing numerous measures.

### Strengths and limitations

There were certain limitations to the current investigation. First, most participants were White and highly educated. As such, more work will be necessary to determine whether the current results are generalizable to more sexual minority groups reflecting greater sociodemographic diversity, particularly given intersectional considerations regarding eating disorder assessment and risk among individuals with multiple marginalized/minoritized identities [47, 47]. Second, as noted above, we examined only cisgender gay men and lesbian women. Although this is preferable to collapsing differing sexual minority identities into a single category, future research should determine whether the brief version of the EDE-Q supported in this study is similarly appropriate for use in other sexual minority groups. Third, although participants were from a range of adult age groups, adolescents were not included in this study. Given that adolescence is an important period in terms of eating disorder risk and sexual identity development [48], future work evaluating whether the current findings replicate in sexual minority adolescents would be helpful. Fourth, future studies will be needed to examine other psychometric properties of the brief EDE-Q that was supported in this study, including test–retest reliability, construct validity, and predictive validity. However, we note several strengths of the current study. First, although there has been recent progress in the field, eating disorder research on cisgender gay men and cisgender lesbian women is still limited. Understanding the appropriateness of assessment measures for use among individuals with these sexual minority identities is important for supporting future work in this area. Second, our comparative CFA approach focused on examining numerous EDE-Q models that have received support in a variety of samples and was followed by a measurement invariance analysis to determine whether the best-fitting model showed invariance across cisgender gay men and lesbian women. Third, the sample sizes of the current study were large and included individuals recruited from around the US, who reflected a wide age range spanning from young to late adulthood.

## Conclusion

The findings from this investigation of cisgender gay men and cisgender lesbian women provide support for a seven-item, three-factor version of the EDE-Q [14] that has previously been supported in studies of multiple populations, including cisgender sexual minority (orientations combined into one group) men and women [22] and gender minority individuals (i.e., transgender men, transgender women, gender-expansive people) [19]. The three subscales demonstrated good internal consistency in both groups, and measurement invariance across gay men and lesbian women was supported at all levels. As this study was conducted with a non-clinical sample, future work should evaluate the performance of this brief version of the EDE-Q in clinical samples of cisgender sexual minority adults with eating disorders. Similarly, additional studies will be needed to empirically derive cut-off scores for sexual minority groups that indicate potential clinically significant elevations in eating disorder symptoms. Finally, given the sociodemographic limitations of the current sample and the potential impacts of intersectionality on eating disorder symptoms, risk, and assessment, there is a strong need for further research that includes sexual minority samples with broader racial, ethnic, and socioeconomic representation.

## Data Availability

Data from The PRIDE Study may be accessed through an Ancillary Study application (details at pridestudy.org/collaborate).

## Abbreviations

AIC: Akaike information criterion
CFAs: Confirmatory factor analyses
CFI: Comparative fit index
ECVI: Expected cross validation index
EDE-Q: Eating disorder examination-questionnaire
LGBTQ: Lesbian, gay, bisexual, transgender, and queer
PRIDE: The population research in identity and disparities for equality study
RMSEA: Root mean square error of approximation
SRMR: Standardized root mean square residual
WLSMV: Weighted least squares estimation method
